# Cochlear implantation impact on health service utilisation and social outcomes: a systematic review

**DOI:** 10.1186/s12913-023-09900-y

**Published:** 2023-08-30

**Authors:** Tolesa Bekele Okuba, Reidar P. Lystad, Isabelle Boisvert, Anne McMaugh, Robyn Cantle Moore, Ramya Walsan, Rebecca J. Mitchell

**Affiliations:** 1 Australian Institute of Health Innovation, Macquarie University, Sydney, New South Wales Australia; 2 Sydney School of Health Sciences, Faculty of Medicine and Health, University of Sydney, Sydney, Australia; 3 Macquarie School of Education, Faculty of Arts, Macquarie University, Sydney, Australia; 4 NextSense Institute, Sydney, Australia

**Keywords:** Cochlear implants, Health service utilisation, Social outcomes, Adults

## Abstract

**Background:**

Hearing loss can have a negative impact on individuals’ health and engagement with social activities. Integrated approaches that tackle barriers and social outcomes could mitigate some of these effects for cochlear implants (CI) users. This review aims to synthesise the evidence of the impact of a CI on adults’ health service utilisation and social outcomes.

**Methods:**

Five databases (MEDLINE, Scopus, ERIC, CINAHL and PsychINFO) were searched from 1st January 2000 to 16 January 2023 and May 2023. Articles that reported on health service utilisation or social outcomes post-CI in adults aged ≥ 18 years were included. Health service utilisation includes hospital admissions, emergency department (ED) presentations, general practitioner (GP) visits, CI revision surgery and pharmaceutical use. Social outcomes include education, autonomy, social participation, training, disability, social housing, social welfare benefits, occupation, employment, income level, anxiety, depression, quality of life (QoL), communication and cognition. Searched articles were screened in two stages ––– by going through the title and abstract then full text. Information extracted from the included studies was narratively synthesised.

**Results:**

There were 44 studies included in this review, with 20 (45.5%) cohort studies, 18 (40.9%) cross-sectional and six (13.6%) qualitative studies. Nine studies (20.5%) reported on health service utilisation and 35 (79.5%) on social outcomes. Five out of nine studies showed benefits of CI in improving adults’ health service utilisation including reduced use of prescription medication, reduced number of surgical and audiological visits. Most of the studies 27 (77.1%) revealed improvements for at least one social outcome, such as work or employment 18 (85.7%), social participation 14 (93.3%), autonomy 8 (88.9%), education (all nine studies), perceived hearing disability (five out of six studies) and income (all three studies) post-CI. None of the included studies had a low risk of bias.

**Conclusions:**

This review identified beneficial impacts of CI in improving adults’ health service utilisation and social outcomes. Improvement in hearing enhanced social interactions and working lives. There is a need for large scale, well-designed epidemiological studies examining health and social outcomes post-CI.

**Supplementary Information:**

The online version contains supplementary material available at 10.1186/s12913-023-09900-y.

## Background

Hearing loss can have a negative impact on an adults’ health and social well-being. Globally, in 2019, an estimated 1.57 billion people had some form of hearing loss, and this figure is estimated to increase to 2.45 billion by the year 2050 [1]. Hearing loss can lead to more frequent use of inpatient or outpatient healthcare services [2, 3], increased fall risk in healthcare facilities [4] and other locations [5], poor communication with providers when using healthcare services [6] that can affect a health consumer’s satisfaction [7] with healthcare delivery and utilisation [8]. Hearing loss is also associated with negative social outcomes, such as reduced academic performance, lower chance of progressing to higher education or undertaking training [9–11], unemployment [10, 11], poor personal relationships [12], feelings of inadequacy and low self-esteem [12, 13], social isolation and loneliness [14], and higher rates of depression and low QoL [15–17]. Integrated approaches that tackle barriers and social outcomes could mitigate some of these effects for both CI users and their health providers [18]. For example, strategies such as noise reduction approaches, and acting on hearing healthcare, are all likely to ameliorate some of the negative aspects of hearing loss [1, 3, 19].

Cochlear implantation is the surgical insertion of an electrode within the inner most part of the ear to transmit sounds from an externally worn device. Cochlear implants are suitable for individuals with a severe to profound sensorineural hearing loss, who do not benefit from standard hearing aids [20, 21]. A cochlear implanted device can improve a person’s ability to understand speech through providing improved access to speech sounds [22–24]. Compared to their preoperative hearing with the use of hearing aids, a majority of CI users demonstrate improvement in speech recognition [25, 26]; however, the magnitude of improvement varies considerably across individual CI users [27–30]. Factors contributing to this variation are unclear, but different studies have suggested that this could be influenced by: age at implantation, age at onset of hearing loss, duration of implant use (up to 1–2 years), duration and cause of hearing loss, placement of implant in the cochlea, integrity of the cochlear nerve, as well as learning ability of the individual living with hearing loss [27, 31, 32].

Several studies have measured hearing outcomes in CI users [25, 33–35]. However, the potential impact of CI on health service utilisation and social outcomes are less understood. To our knowledge, no review has been found that comprehensively synthesized impacts of CI on health service utilisation such as hospital admissions, ED presentations, GP visits and prescription medication use. In addition, there have been few scoping reviews that have examined social outcomes such as work, autonomy and participation post-CI [36]. This review offers some important insights into improve service delivery, health service use and social outcome trajectories of the CI users. Therefore, this systematic review aimed to synthesise the evidence of the impact of CI on health service utilisation and social outcomes in adult CI users.

## Methods

### Eligibility criteria

This systematic review adhered to the Preferred Reporting Items for Systematic Review and Meta-analyses (PRISMA) statement [37] and the protocol was registered with PROSPERO (CRD42023392131). This review included studies reporting on health service utilisation or social outcomes of adults aged ≥ 18 years with considerable hearing loss who received a CI. The comparison was performed either between CI users and non-users with hearing loss but not implanted or within CI users, based on their pre- and post-operative outcomes. Hearing loss was defined according to the World Health Organization’s (WHO) definition into moderate: 41 to 60 dB, severe: 61 to 80 dB, profound or complete deafness: ≥ 81 dB [38, 39]. CI users could be fitted either unilaterally, bilaterally, bimodally (using a hearing aid on the other ear) or by electric-acoustic stimulation (only part of the cochlea is stimulated with a cochlear implant). Articles were excluded if they solely reported on children or individuals with prelingual hearing loss and did not separately report results for adults. Articles were excluded if they were reviews, editorials or opinion pieces, single case report, study protocols, or conference abstracts. This review included English-language articles that were published in a peer-review journal.

The main outcomes of interest are health service utilisation (e.g., hospital admissions, ED presentations, GP visits, CI revision surgery and pharmaceutical use); and social outcomes (e.g., circumstances relating to education, autonomy, social participation, training, disability, social housing, social welfare benefits, occupation, employment, income level, anxiety, depression, QoL, communication abilities and cognition).

Health service utilisation or social outcomes could either be in the short (e.g., < 6 months), medium (e.g., 6–12 months), or long-term (e.g., > 12 months). Health service utilisation included hospital admissions, ED presentations, GP visits, and prescription medication use. Social outcomes included information or circumstances relating to education, autonomy, social participation, training, disability, social welfare (e.g., social housing, welfare benefits), occupation, employment, or income level. Autonomy or independence was defined as the capability of living the way an individual wished to without being reliant on a third person to control, cope and make personal decisions on their life [36, 40]. Whereas the capability of participating in different social situations or activities without limitation due to hearing loss was defined as social participation [36, 41].

### Study selection process

Five databases were searched, including MEDLINE and PsycINFO using the Ovid portal, Scopus, CINAHL using the EBSCOhost portal, and ERIC using the ProQuest portal. The search was conducted from 1 January 2000 to 16 January 2023 for the four common databases. On the recommendation of an educational expert, we added a search of the ERIC database in May 2023. The search strategy was developed in consultation with a university librarian. The full search strategy is provided (see Additional file 1). Snowballing of reference lists from the articles was conducted to identify any potential articles not previously identified. Title and abstract screening involved importing the title, abstract and citation information for each article identified during the database searching into EndNote X20. Duplicates were removed using EndNote. Following duplicate removal, the titles and abstracts of identified articles were screened and assessed for inclusion. Abstracts were excluded if they did not report on health service utilisation or social outcomes post-CI in adults. Uncertainties regarding the inclusion or exclusion of articles based on title and abstract were discussed and consensus was obtained. Full-text screening was done by assessing each article against inclusion criteria. Following the full-text screening, a data extraction form was created and tested on five studies.

### Data extraction

For studies that met the inclusion criteria, key characteristics of each study were extracted, including: authors and publication year; study objective or aim; study type; country/study setting and data collection timeframe; study population (e.g., mean age, sex, and sample size); information on health service utilisation and social outcomes. Data extraction was initially performed by one reviewer and subsequently verified for accuracy by two reviewers and any disagreements were discussed between reviewers and consensus was obtained.

### Data synthesis

Information extracted from the included studies was narratively synthesised by one reviewer and appraised by two reviewers. The narrative synthesis involved tabulating and summarising health service utilisation and social outcomes.

### Quality assessment

The methodological quality of articles was assessed using the Critical Appraisal Skills Programme (CASP) cohort [42] or qualitative [43] study checklists as applicable. Quality assessment was initially performed by one reviewer and independently verified by two reviewers. Any disagreement regarding methodological quality were discussed between reviewers. The checklist consists of 12 questions for cohort, 10 questions for each cross-sectional and qualitative studies. Responses were recorded for each question based on the level of adequate information provided at design and analysis stages in the study. For example, “Was the outcome accurately measured to minimise bias?” ‘Yes’ was recorded for adequate information by looking for measurement or classification bias such as the use of subjective or objective measurements, do the measurement accurately measured what they intend to measure (validated vs. unvalidated tools), reliable method for detecting all cases, similarity of measurement methods in different groups and blinding of outcome assessor or subjects to exposure. Whereas ‘No’ was recorded for missing of one or more information and ‘Can’t tell’ was recorded if the information sought is not applicable. The overall quality of studies was judged based on ‘Yes’ or ‘No’ response to questions regarding relevance, reliability, validity and applicability.

## Results

### Description of studies

**Fig. 1:**
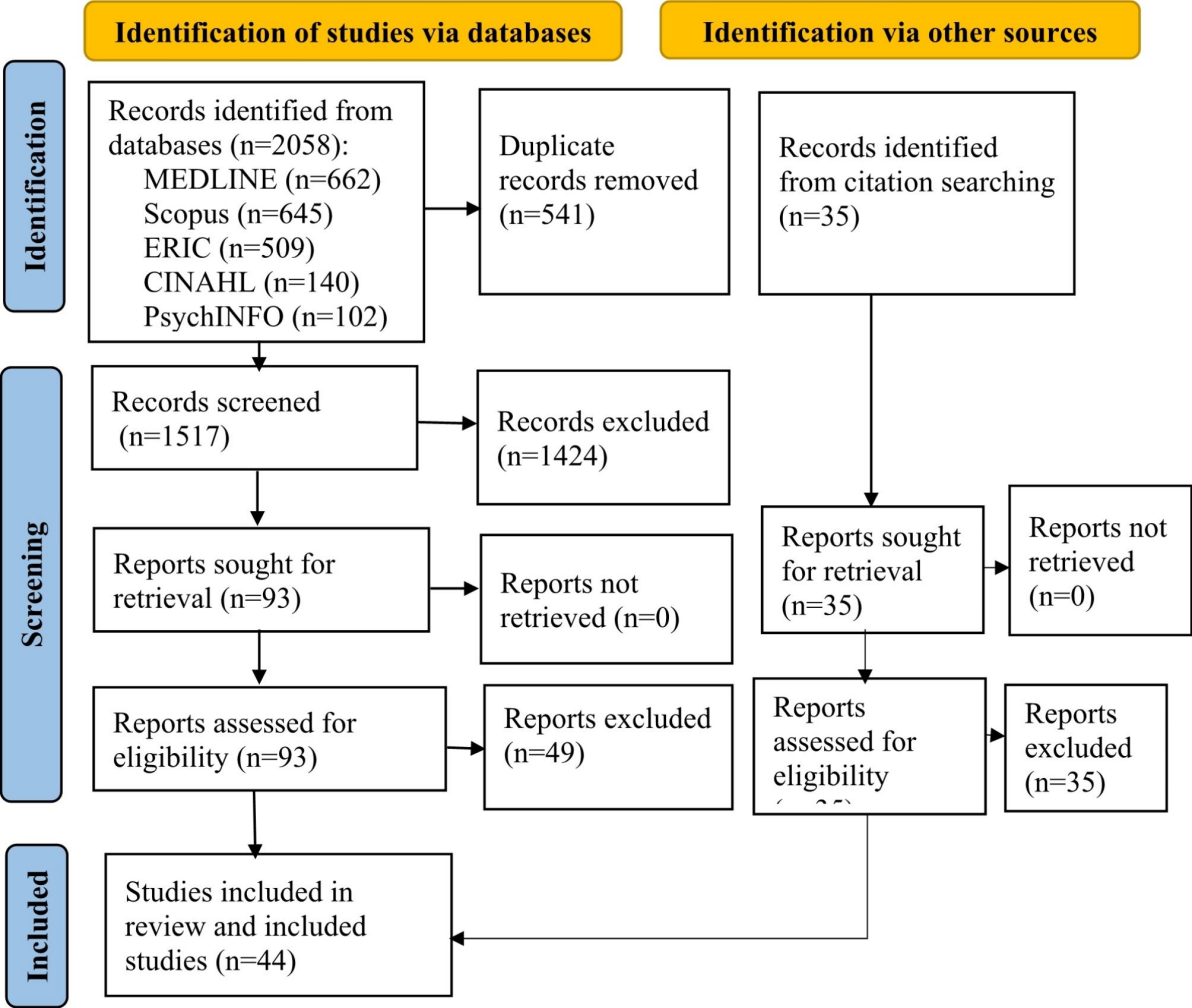
PRISMA flow diagram

The search identified 2093 articles through searching of electronic databases and snowballing search methods. There were 2058 articles identified via the five databases, 541 were removed as duplicates. 1517 articles underwent title and abstract screening. Based on titles and abstracts, 1424 articles were excluded due to not meeting the inclusion criteria. The full text of the remaining 93 articles were assessed for eligibility. Following full text review, 49 articles were excluded due to an irrelevant target population or absence of the primary outcomes. The remaining 44 articles were intensively appraised and met the inclusion criteria (Fig. 1).

There were 20 (45.5%) cohort studies (11 prospective and 9 retrospective), 18 (40.9%) cross-sectional studies and six (13.6%) qualitative studies. Almost all (93.2%, n = 41) of the included studies were conducted in high-income countries. Nine (20.5%) studies were conducted in United States of America (USA), six (13.6%) conducted in New Zealand, four (9.1%) conducted in Canada, three (6.8%) studies each were conducted in Germany and Norway, two (4.5%) studies each were conducted in Australia, Belgium, China, Finland, France, Norway and Poland and one study each was conducted in Brazil, Denmark, Netherlands, Saudi Arabia, Spain, Scotland, Sweden and the United Kingdom (UK). The sample size ranged from 125 to 5,130 participants for health service utilisation and 6 to 637 participants for social outcomes.

All studies included adults with severe to profound post-lingual hearing loss. Three studies [44–46] also included a few participants with prelingual hearing loss but were predominantly focussed on post-lingual hearing loss. Participants implanted with unilateral, bilateral, or bimodal CI were assessed within all studies. The mean duration of hearing loss was not often recorded in the included studies. Where reported (n = 11), the mean duration ranged from 2.5 to 32.7 years. The mean duration of follow-up from the date of CI ranged from 3 months to 23 years. The age of participants ranged from 18 to 101 years at the time of the study. However, one study had one CI user aged 17 years and thus was included in the sample [47]. The mean age of participants was not recorded in nine studies (20.5%), but where recorded, ranged from 21.9 to 80.9 years. Studies assessed outcomes by comparing CI users and non-users with hearing loss but not implanted (18.2%, n = 8) or within CI users, based on their pre- and post-operative outcomes (81.8%, n = 36).

### Health service utilisation

Nine studies (20.5%) reported on health service utilisation (Table 1). Type of health service utilisation examined in each study varied and included readmission for CI revision (n = 5), post-operative surgical and audiological visits (n = 3), pneumococcal vaccination uptake (n = 1), extended hospital length of stay (n = 1), non-home destinations post-CI surgery (n = 1), and medication use (n = 1) (Table 2). The rate of CI revision surgery (with or without reimplantation) ranged from 1.1 to 3.8%. The average time from first implant to revision surgery ranged from 29 months to 7.8 years. Almost all studies indicated an ongoing need for CI revision surgery by users who had experienced complications post-CI. Where complications were reported, soft or hard device failure (n = 5) and flap skin infection, surgical or falls (n = 4) were the main reasons for the revision surgery. The mean number of health service visits ranged from 1.9 to 4.3 times a year. For example, one study [48] compared the health service visits of older (≥ 80 years) and younger adults (aged 60–79 years) within the CI group and found no difference in number of visits between the age groups, with the average number of visits decreasing for both age groups in the second year of post-operative follow-up. Pneumococcal vaccination uptake increased in CI users after a follow-up reminder in another study [49]. The study that examined medication use, identified that prescribed medication was used on average for 1.8 illnesses among CI users and for 3.1 illnesses in a non-user group [50].

**Table 1:** Characteristics of included studies

Authors and publication year	Study method	Country and study setting	Data collection, follow-up or surgery date	Study population (mean age, sex sample size)	Health service utilisation	Social outcomes
Aldhafeeri et al., 2021 [90]	Retrospective cohort	Saudi Arabia, hospital	CI surgery between January 2011 and July 2017	Adults with CI (mean age = 43.6 years, n = 102).	✓	
Carpenter et al., 2010 [49]	Cross-sectional	USA, hospital	Follow-up post-CI during July 2007 to August 2008	Adults aged ≥ 18 years with CI (n = 125).	✓	
Chapman et al., 2017 [51]	Cross-sectional	Denmark, online survey	Survey conducted in 2014	Adults aged < 26 and > 25 years of age (mean ± standard deviation (SD) = 43.1 ±14.23 years, n = 254 with CI and n = 547 without CI) with moderate to severe hearing loss.		✓
Chen et al., 2022 [91]	Retrospective cohort	China, hospital	CI revision surgery during 1996 to 2019	Adults with CI (age at implantation ≥ 18 years, n = 929).	✓	
Claes et al., 2018 [62]	Prospective cohort	Belgium, hospital	NR, follow-up 1 year post-CI	Older adults with post-lingual severe hearing loss and with CI (median age = 71.5 years, n = 20, men = 12 and women = 8).		✓
Clinkard et al., 2015 [57]	Cross-sectional	Canada, CI programme	NR	Adults with CI (mean age = 52.1 years, n = 65, men = 28 and women = 32, missing = 5) who received health services in a large urban setting.		✓
Cole et al., 2022 [97]	Cross-sectional	USA, multicentre national database	CI surgery between 2001 to 2018	Adults aged ≥ 18 years (median age = 60 years, n = 5130, men = 2385 and women = 2745).	✓	
Czerniejewska-Wolska et al., 2015 [68]	Prospective cohort	Poland, 10 CI sites	NR	Adults aged ≥ 60 years with CI (mean age = 67.8 years, n = 20, men = 12, women = 8).		✓
Fazel et al., 2007 [98]	Cross-sectional	UK, hospital	Received a CI between 1988 and 2001	Adults with CI (mean age = 45 years, n = 65, men = 39, women = 26).		✓
Fitzpatrick et al., 2022 [103]	Qualitative	Canada, hospital	Received a CI between 2002 and 2013	Adults aged ≥ 18 years and unilaterally implanted (mean ± SD age = 55.9 ± 17.1 years, n = 8 CI users (men = 2 and women = 6) and men coaches = 2, women coaches = 2 who completed a 24-week auditory-verbal intervention program for 1 h per week.		✓
Goh et al., 2016 [44]	Cross-sectional	New Zealand, Southern Cochlear Implant Programme (SCIP)	Received a CI between 1994 and 2011	Children who received their CI before 19 years of age but aged ≥ 19 years at the time of the survey (mean ± SD age = 23 ± 3.0 years, n = 26 (men = 13 and women = 13) including one non-user.		✓
Guitar et al., 2013 [50]	Cross-sectional	New Zealand, Northern Cochlear Implant Trust (NCIT)	NR	Adults with CI mean age = 58.6 years, n = 119 (men = 57 and women = 61) received a CI or follow-up care at least 12 months ago, and on waiting list mean = 60.2 years, n = 44 (men = 17 and women = 27).	✓	
Gumus et al., 2021 [92]	Retrospective cohort	Turkey, clinic	Received a CI between August 2005 to August 2019	Adults with CI (mean age at first implant = 34.91 years, n = 423 children and adults).	✓	
Harkonen et al., 2017 [73]^**‡**^	Cross-sectional	Finland, hospital	NR	Adults with hybrid CI mean age at implantation was 49 years, n = 8 (men = 3 and women = 5) and with unilateral hybrid CI (n = 6) and bilateral hybrid CI (n = 2).		✓
Harkonen et al., 2015 [75]^**‡**^	Prospective cohort	Finland, hospital	NR	Working adults with unilateral CI (mean age = 41 years, n = 15, men = 6 and women = 9).		✓
Hawthorne et al., 2004 [76]	Prospective cohort	Australia and New Zealand, two CI clinics	NR, data were collected for 2.5 years	Nine adults from Australia and 25 from New Zealand (mean ± SD age = 49 ± 13 years, n = 34, men = 16 and women = 18).		✓
Hixon et al., 2017 [72]	Cross-sectional	USA, hospital	Received active care from 1989 to unspecified date	Adult > 18 years with CI (mean age = 61.6 years rural and 62.4 years urban, n = 91, men = 42 and women = 49 (n = 32 from urban counties, n = 26 from moderately rural counties, and n = 33 for extremely rural counties).		✓
Hogan et al., 2001 [52]	Cross-sectional	Australia and New Zealand, three metropolitan CI clinics	NR	148 (men = 69 and women = 79) with CI and 54 (men = 28 and women = 26) without CI and their partner (n = 136).		✓
Hogan et al., 2002 [104]	Qualitative	New Zealand, two centres	NR	Adults with CI (aged 23 to 60 years, n = 12, men = 5 and women = 7).		✓
Huarte et al., 2017 [93]	Retrospective cohort	Spain, clinic	Survey of hearing loss during September 2013 and September 2014	Adults with bilateral CI and profound hearing loss (mean age = 47.92 years, n = 60, men = 34 and women = 26).		✓
Issing et al., 2022 [69] ^**‡**^	Prospective cohort	Germany, hospital	Survey post-CI from the 1st quarter of 2017 until the 4th quarter of 2017	Older adults ≥ 65 with unilateral CI for the first time between for at least one and a maximum of 10 years prior to survey (mean ± SD age at the survey = 75.3 ± 7.3 years, n = 84, men = 36 and women = 48).		✓
Issing et al., 2020 [67] ^**‡**^	Prospective cohort	Germany, hospital	Survey of hearing loss during 3rd quarter of 2015 to 3rd quarter of 2017	Adults ≥ 65 years with CI (mean age = 73.5 ± 4.9 years, n = 34, men = 13 and women = 21).		✓
Kay-Rivest et al., 2022 [94]	Prospective cohort	USA, hospital	Received CI between February 2021 to April 2022	Adults with CI ≥ 65 years (mean ± SD age = 80.9 ± 7.4 years, n = 46, men = 29 and women = 17), frail (n = 5), prefrail (n = 10) and not frail (n = 31).	✓	
Kos et al., 2007 [55]	Cross-sectional	Switzerland, hospital	Survey post-CI in 2005	Adults ≥ 18 years (mean ± SD age = 56 ± 13 years, n = 60, men = 31 and women = 29) who received a multi-channel device.		✓
Krabbe et al., 2000 [99]	Cross-sectional	Netherlands, NR	Survey in 1998	Adults with CI (mean ± SD age = 50 ± 16 years, n = 45, men = 21 and women = 24) and on waiting list (mean ± SD age = 51 ± 16 years, n = 46, men = 28 and women = 18).		✓
Lachowska et al., 2013 [87]	Retrospective cohort	Poland, Hearing Implant Centre	NR	Older adults with CI (mean ± SD age = 72.4 ± 8.1 years, n = 31).		✓
Looi et al., 2011 [74]	Cross-sectional	New Zealand, CI Programme	NR	Adults mean ± SD age at the time of study = 56.6 ± 14.5 years, with CI (n = 94, men = 36 and women = 58) and on waiting list, mean age = 56.5 ± 15.3 years (n = 70, men = 32 and women = 38).		✓
Maki-Torkko et al., 2015 [105]	Qualitative	Sweden, hospital	Survey post-CI between Nov 2008 and April 2011	Adults ≥ 18 years and unilateral implants between Feb 1992 and Jan 2010 (mean ± SD age = 66.0 ± 14.3 years, n = 101, men = 40 and women = 61).		✓
Marschark et al., 2018 [100]	Cross-sectional	USA, Rochester Institute of Technology (RIT)	NR	115 with hearing loss (with CI = 49) and 80 hearing university students.		✓
Mertens et al., 2021 [61]	Prospective cohort	Australia, Belgium, Spain, Poland and UK, hospitals	Test battery post-CI between April 2015 to August 2019	Older adults ≥ 55 years, mean ± SD age = 72 ± 7 years with CI (n = 24) and mean age = 73 ± 9 years without CI (n = 24).		✓
Mo et al., 2004 [59]	Cross-sectional	Norway, hospital	Received a CI between 1986 to 2000	Adults ≥ 18 years with CI (mean ± SD age = 54.4 ± 16.7 years, n = 84, men = 40 and women = 44), with HA (mean ± SD age = 56 ± 19.0 years, n = 60, men = 31 and women = 29), non-CI group (mean ± SD age = 50.7 ± 15.7 years, n = 35, men = 17 and women = 18). Three users who received CI as children but > 18 years of age at the time of study were included.		✓
Mo et al., 2005 [60]	Prospective cohort	Norway, hospital	NR	Adults with CI (mean ± SD age = 57.6 ± 14.5 years, n = 27, men = 12 and women = 15).		✓
Monteiro et al., 2012 [58]	Retrospective cohort	Canada, hospital	Received a CI between 1984 to 2009	Adults ≥ 18 years with CI (n = 637).		✓
O’Neill et al., 2021 [101]	Cross-sectional	USA, NR	NR, data were collected prior to the onset of the COVID-19 pandemic	Adults with CI (mean ± SD age = 62.3 ± 9.5 years, n = 18, men = 4 and women = 14).		✓
Park et al., 2011 [95]	Retrospective cohort	Canada, CI Program	Received a CI between 2000 to 2009	Adults with CI (mean ± SD age = 56 ± 15 years, n = 161, men = 62 and women = 99).		✓
Raymond et al., 2020 [48]	Retrospective cohort	USA, hospital	Received a CI between 1987 and 2018	Older adults > 59 years with CI (mean age = 71.5 years, n = 59, men = 29 and women = 30).	✓	
Rember et al., 2009 [56]	Qualitative	Norway, hospital	Received a CI between 2000 and 2006	Adults with unilateral CI (mean ± SD age = 56.2 ± 15.2 years, n = 74, men = 30 and women = 44).		✓
Ross et al., 2007 [45]	Qualitative	Scotland, CI centre	NR	Adults with CI and their partner (mean age = 67 years, n = 6, men = 3 and women = 3).		✓
Saxon et al., 2001 [102]	Cross-sectional	USA, CI programme	NR	Adults with CI (n = 13) and work supervisors (n = 9).		✓
Sonnet et al., 2017 [53]	Prospective cohort	France, hospital	Received a CI between Jan 2014 and Oct 2016	Adults ≥ 65 years with CI (mean age = 72.5 years, n = 16, men = 6 and women = 10).		✓
Sorrentino et al., 2009 [96]	Retrospective cohort	France, hospital	NR	Adults with CI (mean age at implantation = 44 years, n = 286).	✓	
Spencer et al., 2012 [47]	Cross-sectional	USA, hospital, and clinic	Received a CI between 1987 and 1999	Adults with CI (mean ± SD age = 21.9 ± 3.8 years, n = 41, men = 19 and women = 22).		✓
Vieira et al., 2018 [46]	Qualitative	Brazil, Universidade Federal de São Paulo (UNIFESP)	NR	Adults with CI (mean age = 41.7 years, n = 16, men = 11 and women = 5).		✓
Volter et al., 2018 [54]	Prospective cohort	Germany, hospital	Received a CI between 2016 and 2017	Adults with CI (mean ± SD age = 65.8 ± 8.9 years, n = 60).		✓

**‡**=There might be some overlap in the participants in the two Harkonen et al. and Issing et al. papers; CI = Cochlear implantation/implant; NR = Not reported

**Table 2:** Characteristics of included studies that examined health service utilisation of CI users

Authors and publication year	Objective or aim	Health service utilisation measures	Key findings
Aldhafeeri et al., 2021 [90]	To discuss experience of managing cochlear implant cases that required revision surgery.	CI revision surgery (with or without reimplantation).	Overall, four CI revision surgeries were performed. The main reasons for revision were device failure, surgical (misplaced) or medical (infection) related.
Chen et al., 2022 [91]	To determine factors related to need for cochlear implant revision surgery, to identify the rate of revision surgery, and to elucidate the cumulative survival and device survival in different age groups.	CI revision surgery.	Of the 929 CI users aged ≥ 18 years, 10 CI revision surgeries were conducted. A revision rate of 1.1%. Three device failures (n = 2 hard and n = 1 soft failure) and seven non-device (i.e., n = 3 electrode displacements; n = 1 infection; n = 1 mis-insertion; n = 1 device migration; n = 1 facial paralysis) were the reasons for the revision. The mean ± SD for 5-year cumulative and device survival rates were 98.7 ± 0.4% and 99.5 ± 0.3%, respectively.
Carpenter et al., 2010 [49]	To increase vaccination rates for bacterial meningitis using information dissemination through brochure and electronic media, and ongoing reminder for CI users.	Pneumococcal vaccination utilisation was collected using ongoing mailed, email, phone call and patients’ medical record review.	Pneumococcal vaccination rates increased from 49–99% following CI. Majority of CI users received vaccination only after the follow-up reminder.
Cole et al., 2022 [97]	To determine the association of chronological age and frailty as measured by 5- and 11-factor modified frailty index (mFI-5, mFI-11) on post-operative outcomes of participants undergoing CI.	Extended hospital length of stay (i.e., > 75th percentile of study population) average and standard deviation in days and non-home discharge destinations post-CI using the Modified Frailty Index (mFI-5 and mFI-11) [106].	Increased frailty of CI users likely associated with extended length of hospital stay and non-home discharges. Of the 5130 CI users, 320 (6.2%) were discharged to a non-home destination, such as aged care centre.
Guitar et al., 2013 [50]	To determine whether people on a waiting list for CI are more likely than those who have a CI to suffer from illnesses which are potentially mediated by stress.	Number of physician visits and medication use in the past year were assessed using 21-item Depression, Anxiety and Stress Scale (DASS-21) [107], the Short-form health questionnaire (SF-36) [108], and self-rated dissatisfaction with hearing.	Time since implantation was 5.73 years (range 375–6653 days) for CI group. Individuals on the waiting list waited on average for 18 months (range 45-1960 days). Participants on waiting list visited a physician on average 6.2 times (SD ± 4.8) a year while participants with CI visited 4.3 times (SD ± 3.7). Participants on the waiting list took prescription medication on average for 3.1 illnesses (SD ± 2.4) while those with a CI took prescription medication on average for 1.8 illnesses (SD ± 1.9). People on the waiting list were more likely to take prescription medication for migraines, ear infections, and sleep disturbance compared to people with a CI. The overall psychological distress, specifically anxiety and stress were higher in the waiting list group compared to people with a CI.
Gumus et al., 2021 [92]	To determine reasons for CI revision surgeries in paediatric and adult groups.	CI revision surgery	Overall, six CI revision surgeries were performed, and the revision surgery rate was 1.4%. Three device failures (i.e., hard failures (n = 2), and soft failure due to voice problem (n = 1). Three medical-related problems (i.e., flap skin infections (n = 2) and chronic otitis media (n = 1)).
Kay-Rivest et al., 2022 [94]	To evaluate the frailty phenotype in a population of older adults and determine the association of frailty with (i) preoperative complications, (ii) need for vestibular rehabilitations after surgery, and (iii) early speech perception outcomes.	(1) Post-operative vestibular/aural rehabilitation post-CI assessed by the Fried Frailty Index [109] and patients’ medical records. (2) CI revision	There were 10 pre-frail, 5 frail, and 31 non-frail users. (1) The number of missed follow-up visits (combined surgeon, audiologist, speech language pathologist visits) was higher for frail patients (n = 7 visits; range 1–10 visits) compared to pre-frail (n = 3 visits; range 0–4 visits) and non-frail (n = 2 visits; range 0–5 visits) users. (2) Four users developed vertigo, three users required vestibular rehabilitation, one user had a post-operative fall, and one had complication that required implant revision.
Raymond et al., 2020 [48]	To determine the association between geriatric age and post-operative healthcare utilisation post-CI.	Post-operative surgical and audiological visit rates up to 2 years post-CI, along with phone calls to the otology and audiology departments recorded in the electronic health record (eMR) after their surgery related to their CI surgery or implanted device using patients’ medical record.	The mean duration of hearing loss was 25.4 years, and the mean duration of follow-up post-CI was 37 months. In the first post-operative year, there was 1.9 ± 1 mean ± SD number of surgical visit for 60 to 69 years old, 1.9 ± 0.8 visits for 70 to 79 years old and 2 ± 1.8 visits for individuals aged ≥ 80 years and the mean number of audiological visits was 5.4 ± 1.5 for 60 to 69 years old, 5.5 ± 0.7 for 70 to 79 years old and 5.6 ± 0.7 for individuals aged ≥ 80 years. The number of phone calls in the first post-operative year were 0.9 ± 1.4 for 60- to 69-year-olds, 1.7 ± 2.5 for 70 to 79 years old and 1.2 ± 1.2 for individuals aged ≥ 80 years old. In the second post-operative year, the mean numbers of surgical visits decreased by 0.12 ± 4.3, 0.17 ± 3.8, and 0 by each age group and audiology visits in the second year were 1.4 ± 1.4, 1.3 ± 0.9, 1 ± 1.3 by age group, respectively. The number of phone calls in the second post-operative year were 0.3 ± 0.5 for 60 to 69 years old, 0.5 ± 0.8 for 70 to 79 years old and 0.1 ± 0.3 for individuals aged ≥ 80 years. There was no significant difference in health service utilisation between the age groups.
Sorrentino et al., 2016 [96]	To evaluate cochlear implant revision surgery experience and to compare with available literature.	CI revision surgery	Of 286 CI users originally with profound sensorineural hearing loss, 11 (3.8%) adults underwent CI revision surgery, with two adults undergoing reimplantation twice. Device failure including hard failure (n = 7) and soft failure (n = 4), and medical-related (n = 9) were reported as the main reasons for revising surgery. Four adults had revision surgery due to a flap skin infection.

### Social outcomes

Thirty-five (79.5%) studies reported on social outcomes, 14 (40.0%) were cohort, 15 (42.9%) were cross-sectional and six (17.1%) were qualitative studies. The majority (71.4%, n = 25) of these studies reported social outcomes as a primary objective of the study (Table 3). Of the 35 studies, 21 (60.0%) reported on work or employment, 15 (42.9%) on social participation, nine (25.7%) on autonomy or independence, eight (22.9%) on education, six (17.1%) on perceived hearing disability, three (8.6%) on income, and two (5.7%) on safety and welfare.

**Table 3:** Characteristics of included studies that examined social outcomes of CI users

Authors and publication year	Objective or aim	Social outcome measures	Key findings
Chapman et al., 2017 [51]	To determine the role of having or not having a CI in relation to deaf identity and social participation.	(1) Perceived hearing disability: measured by perceived experiences of being a deaf, such as feeling of being discriminated against, feeling limited, and challenges working with hearing peers. (2) Identity: measured by the Deaf Identity Development Scale (DIDS) [110] and Deaf Acculturation Scale (DAS) [111] and reported as a deaf, hearing, bicultural and marginal identity. (3) Social participation: hearing and deaf friendship and engagement in forms of social activities, such as frequency of meeting with deaf and hearing friends, participating in deaf cultural events and meeting in mainstream organisations (i.e., unions, sports, political, and housing).	Overall, 30.3% had at least one CI and the mean duration since implant was 36.8 (SD ± 17.9) years. (1) No difference was observed among the CI users in terms of feeling discriminated against because of hearing disability. However, those with marginal identity reported higher levels of feeling discriminated against compared with the three identity categories (i.e., deaf, hearing, and bicultural). Challenges working with hearing peers: The CI group were less likely to report cultural differences as a challenge than people without a CI (3.1% vs. 7.6%). People aged > 25 years with a CI reported less cultural differences at work compared to people without a CI (3.0% vs. 8.1%). (2) Comparing participants with and without a CI, those without a CI were more likely reported a deaf identity (40.9%, n = 204), whereas those with a CI were more likely to report a hearing identity (41.1%, n = 9). A higher proportion of people aged > 25 years with a CI reported a hearing identity than people without a CI (46.3% vs. 39.0%) and a lower proportion of people aged > 25 years reported a deaf identity compared to people without a CI (11.7% vs. 18.0%). There was no significant difference for hearing or deaf identity for people aged < 26 years. (3) The CI group socialized more with hearing friends than the without CI group (11.8% vs. 10.0%).
Claes et al., 2018 [62]	To evaluate cognitive change in severely hearing-impaired older adults post-CI.	Assessments were performed pre-CI and at 6 and 12 months post-CI. (1) Cognition: measured by the Assessment of Neuropsychological Status for Hearing-impaired individuals (RBANS-H) [112] over five cognitive domains: immediate memory (i.e., listing learning and story memory), visuospatial or constructional (i.e., copying geometric figures, and line orientation to identify matching lines), language, (i.e., picture naming and sematic fluency), attention (i.e., digit span and coding) and delayed memory (i.e., list recall, recognition, story recall and figure recall). (2) HRQoL: assessed by Nijmegen Cochlear Implant Questionnaire (NCIQ) [113] using three main domains such as physical (sound and speech perceptions), psychological (self-esteem), and social functioning (activity and social interaction). (3) Perceived hearing disability: evaluated by speech, spatial and qualities of hearing including activity limitations and social interaction using Speech, Spatial, and Qualities of hearing Scale-12 (SSQ12) [114]. (4) Anxiety and depression: measured by the Hospital Anxiety and Depression Scale (HADS) [115].	Before CI, 6 (30%) users had bilateral hearing aids, 9 (45%) unilateral, and 5 (25%) did not use any hearing aids. The mean duration of hearing loss before CI was 26.9 years (range 0.3 to 55 years). (1) The mean ± SD of the RBANS-H score was 89.6 ± 15.2 pre-CI and increased to 93 ± 12.8 and 95.3 ± 13.7 at 6 and 12 months post-CI, respectively. There was a significant improvement at 12 months post-CI in the overall cognition score, and immediate memory, attention, and delayed memory subdomains. (2) Overall, the mean ± SD score of HRQoL was 31.8 ± 11.4 pre-CI and increased to 64.3 ± 10.5 at 6 months and 64.0 ± 12.2 at 12 months post-CI. The subdomains (i.e., speech and sound perception) scores increased at 6 months post-CI but there was no additional improvement at 12 months post-CI. (3) Perceived hearing disability significantly improved from pre-CI to 6 months post-CI but there was no further improvement at 12 months post-CI. The pre-CI mean ± SD score of self-perceived hearing disability was 1.4 ± 1.0 and increased to 4.4 ± 1.8 at 6 months and to 4.3 ± 1.5 at 12 months post-CI. (4) Anxiety and depression showed significant improvement from pre-CI at 6 months post-CI but there was no additional change at 12 months post-CI. The mean ± SD score of anxiety was 6.6 ± 3.2 pre-CI, 4.3 ± 2.0 at 6 months, and 5.7 ± 2.2 at 12 months post-CI. While depression pre-CI was 6.9 ± 4.0, decreasing to 4.1 ± 2.6 at 6 months, and 5.2 ± 3.5 at 12 months post-CI.
Clinkard et al., 2015 [57]	To evaluate the extent to which personal income changes in people who receive a CI and to measure the extent to which age, education, hearing score pre-implant and time with implant improves income post-implant.	Income bracket, and type of employment pre- and post-CI at mean ± SD duration of 6.6 ± 4.6 years post-CI.	Of 64 CI users who reported their income bracket, 20 (31%) reported increased level of income > CAD $20,000 (i.e., from $20,000 to $80,000) post-CI. Only one CI user reported a decreased income level post-CI. Younger CI users, aged ≤ 45 years were 75% more likely to report an increase in income post-CI. Employment increased from 40 participants pre-CI to 49 participants post-CI: a 14% increase in employment post-CI.
Czerniejewska-Wolska et al., 2015 [68]	To evaluate the quality of life and quality of hearing after Nucleus CI in patients aged ≥ 60 years.	Four outcomes were measured pre-CI and at 1 year post-CI. (1) Work performance. (2) Employment history. (3) Perceived hearing disability: measured by the SSQ [116] . (4) QoL: measured by the Health Utility Index (HUI) [117] to assess domains related to sight, hearing, speech, emotions, pain, locomotion, fitness, cognitive functions before switch-on of the processor.	(1) Pre-CI, hearing loss was reported to always or sometimes have affected their work. One-year post-CI, of the 20 users, two users reported improvement in their work performance and one user reported no change in their work performance. (2) 14 users were retired; one was a part-time worker, two were full-time workers and information on employment was not reported for 3 users. (3) One-year post-CI, speech understanding increased by 180%, spatial hearing by 135% and quality of hearing increased by 98%. Bimodal hearing (i.e., hearing aid plus CI) use showed more improvements in quality of sounds in 7 of 10 users compared with those using CI only. (4) Overall, QoL before CI was reported as 0.38. That is on 0 (death) to 1 (full health) scale. At 1-year post-CI, the QoL score increased by 33% (i.e., up to 0.5 on the scale). Of all QoL domains, 61% was improvement in perception of hearing and 19% in communication (speech) 1-year post-CI.
Fazel et al., 2007 [98]	To determine the effect of CI on employment and employees’ perception of career opportunities.	(1) Employment: Unemployed users were asked if they believed their unemployment was because of hearing loss. Whereas employed users were asked whether having hearing loss adversely affected their career prospects and whether their career prospects changed post-CI. (2) Job satisfaction: users were asked to rate their job in terms of satisfaction on a scale of 1 to 10 both pre- and post-CI.	(1) Of 65 CI users, 20 (30.8%) were unemployed pre-CI and 11 (16.9%) remained unemployed post-CI. Pre-CI, 12 (60%) of users believed that their hearing disability was a reason for their unemployment. Post-CI, n = 9 previously unemployed users found jobs (n = 6 believed finding a job was due to an improvement in their hearing ability and n = 3 did not feel gaining employment was due to an improvement in their hearing ability). Eleven users remained unemployed and felt it was due to their hearing deficit. Forty-five (69.2%) users were working pre-CI increasing to 54 (83.9%) post-CI. Of the working users, 26 (57.7%) users believed that their hearing disability had affected their career while 18 (40%) believed that a CI substantially improved their career progression. Of 54 users who were working post-CI, 50 (92.6%) believed that increased confidence at work was due to better hearing because of the implant. Nine users were in different job post-CI compared to pre- CI. Seven of the nine users felt they could not have done their new job without the aid of their CI. (2) The mean job satisfaction pre-CI was 5.56 (range 1–10) significantly increasing to 6.82 (range 4–10) post-CI.
Fitzpatrick et al., 2022 [103]	To explore the perspective of CI users and their coaches with an auditory-verbal intervention as an example of implant rehabilitation, and their views on perceived benefits and challenges related to the intervention.	Social participation: information collected by semi-structured focus group discussion for social functioning such as change in confidence of participating in daily living, entertainment, and social activities after 24 weeks pre- and post- auditory-verbal intervention.	The mean ± SD duration of CI use was 4.0 ± 3.6 years. Post-intervention, users showed increased confidence in participating in daily life, entertainment, and social activities. For example, some users had felt more confidence in using the telephone. Several users reported improved communication in speech recognition score after the 24-week intervention.
Goh et al., 2016 [44]	To report the education and vocational achievements and social participation of CI recipients graduating from a paediatric CI programme.	(1) Education: highest qualification achieved and desire to progress to further education measured by the Satisfaction With Life Scale (SWLS) [118, 119]. (2) Employment: job-seeking methods, job satisfaction and career aspirations measured by the SWLS. (3) Social participation: measured by SWLS and Hearing Participation Scale (HPS) [120]. A score of 5 represents low satisfaction and a score of 35 represents high satisfaction.	Of 26 users, only 3 had their CI pre-lingual. Nearly all (96%) users used their CI daily. Of the CI users, 62% had unilateral implants. Nearly all (92%) users used spoken language as their main communication methods and the remaining 8% used sign language. (1) 43% of CI users had a qualification post-secondary school. Of these, 31% had a university degree. Over three quarter (76%) of users had positive opinion that their CI was useful for learning. (2) The majority (88%) of CI users were employed prior to the survey, of which 46% were in full-time and 42% in part-time or casual employment. 50% of the participants received assistance from their family to find a job. There was promising job satisfaction and work relations reported with 66% satisfied with their job and 70% felt they get along with workmates. (3) The mean total score for SWLS was 24.9.
Harkonen et al., 2017 [73]	To assess the effect of hybrid CI on quality of life, quality of hearing and working performance in adults and to compare the long-term results of hybrid CI with adults who of conventional unilateral CI, bilateral CI, and single-sided deafness (SSD) with CI.	Users’ QoL and work performance were assessed at 3.6 years post-CI. (1) QoL (i.e., general, social support and physical health): measured by the Glasgow Benefit Inventory (GBI) [121]. (2) Work performance using questions regarding how the CI aided their work and influenced their career development.	The average time of hearing loss before implant was 2.5 years. The mean time between the hybrid CI and the study was 3.6 years. (1) QoL was improved in all CI users at follow-up. (2) Working performance improved for all CI users. CI had positively influenced participants’ career planning and communication with co-workers and speaking on the telephone
Harkonen et al., 2015 [75]	To evaluate the benefits of sequential bilateral CI in working performance, quality of life, and quality of hearing (QoH).	(1) QoL: pre- and post-CI (6- and 12-months post activation) of a second CI using GBI. (2) Depression: measured by the 15 dimensions (15D) [122]. (3) Work-related stress: measured by the Finnish Occupational Stress Questionnaire and work performance regarding how the second CI aided their work and influenced their career development.	Ten patients used a hearing aid in the contralateral ear before they had their second CI. On average, the first CI had been implanted 4.7 years. (1) The mean ± SD total score of QoL was 43 ± 19 pre- the first CI, 35 ± 19 post 6 months with the second CI and 39 ± 17 post 12 months with the second CI. The mean score for general health was 60 ± 26 pre-CI, 50 ± 25 at 6 months post second CI and 56 ± 27 at 12 months post the second CI. (2) The total mean of 15D score was 0.93 with a single CI and improved to 0.95 and then to 0.96 at 6 and 12 months post the second CI. The dimension of depression improved from 0.84 pre-CI to 0.91 and then to 0.94 post 6 and 12 months with the second CI, respectively. (3) The mean work-related stress score did not significantly change after the second CI but decreased after 1-year post-CI (results not shown). After bilateral CI the users reported managing much better at work and being more alert after their working day. The second CI had a slight positive influence on their career development or planning.
Hawthorne et al., 2004 [76]	To document the HRQoL and social participation benefits of adult patients receiving CIs in Australia and New Zealand.	Assessed pre-CI and 3 and 6 months post-CI. (1) Social participation: measured by the HPS [120]. (2) HRQoL: measured by the Quality of Life (AQoL) [123]. Information was collected for four domains: independent living, social relationship, physical senses, and psychological wellbeing.	At Pre-CI, three-quarter (75%) of users had profound hearing loss and most (44%) had the condition within the last five years and almost all were referred to a CI program. (1) The mean ± SD of HPS score was 0.48 ± 0.15 pre-CI, 0.64 ± 0.16 at 3-month and 0.68 ± 0.18 at 6-month post-CI. Social participation increased by 42% (20 HPS scale points) at 6 months post-CI. (2) The mean ± SD of AQoL utility score was 0.36 ± 0.23 pre-CI, 0.50 ± 0.29 at 3-month and 0.64 ± 0.28 at 6-month post-CI. HRQoL increased by 78% (28 utility points) at 6 months post-CI.
Hixon et al., 2017 [72]	To compare the timing and impact of hearing healthcare of rural and urban adults with severe hearing loss who use CI.	(1) Highest level of education. (2) Income bracket. (3) Self-reported impact of hearing loss on employment.	A total of 64 users had experienced hearing loss for at least 10 years and had progressive hearing loss. The average duration between onset of hearing loss and a CI was 30 years (36 years for rural and 29 years for urban-metro users). (1) 44% of participants reported that they felt that their hearing loss prevented them from completing education and 58% had a desire to complete higher education, which was limited by hearing loss. 39% rural users were pursuing post-high school education compared with 88% of the urban area residents. (2) More than half (54%) of CI users in urban areas had USD $60,000 income per year compared with 9% of rural. (3) 78% of CI users indicated hearing loss caused difficulty in performing their job. 49% of CI users reported that hearing loss prevented their hiring and 30% reported job loss due to their hearing loss. 40% of CI users felt they experienced discrimination in the workplace related to their hearing loss.
Hogan et al., 2001 [52]	To examine the extent to which CI and related rehabilitation improve HRQoL and social participation for deafened adults and their partners.	(1) HRQoL: measured by the AQoL [123]. (2) Social participation: measured by participation scale (PS) [120] including self-esteem, social interaction, and hearing handicap. (3) Autonomy: measured as independent living and collected by using AQoL [123].	The average ± SD time since implantation was 4.9 ± 4.2 years. The duration of hearing loss onset ranged from ≤ 4 to ≥ 20 years. (1) Of the AQoL domains, only physical sense showed significant difference between CI and non-users (0.78 vs. 0.58; p < 0.01). Overall, the mean AQoL utility value was 50% higher for CI than that of non-CI users (0.57 vs. 0.38; p < 0.01). (2) There was a significant difference in the mean overall PS score between CI and non-CI users (3.30 vs. 2.51; p < 0.01), and the PS subscales for self-esteem (3.16 vs. 2.31; p < 0.01), social interaction (3.34 vs. 2.62; p < 0.01), and hearing handicap (3.39 vs. 2.62; p < 0.01). (3) There was no significant difference in independent living between the CI and non-CI users (0.89 vs. 0.87; p < 0.44).
Hogan et al., 2002 [104]	To examines the impact of implant technology on the working lives of CI recipients.	(1) Employment: through narrative from focus group discussion on deafness and CI in the workplace. (2) Job satisfaction: views on the impact of the CI on their work lives.	(1) Compared with pre-CI, users indicated that they were able to find jobs they were trained for with a greater confidence post-CI. Following CI, work experience was reported to be substantially improved. For instance, users felt they were able to take job risks such as looking for better career opportunities. Users also indicated that they developed a greater confidence, friendships, positive interactions, being a part of things, new priorities and achieving. (2) There was a reported improvement in job satisfaction post-CI compared to pre-CI.
Huarte et al., 2017 [93]	To explore the impact of CI on the working life of adults with bilateral severe-profound hearing loss.	(1) Highest level of education. (2) Industry of employment. (3) Job satisfaction: measured as working life satisfaction one year post-CI.	The mean duration of CI use was 9 years. (1) One-third of users had vocational qualification, 16.7% each had primary school, secondary school, or higher vocational training. 11% had a university degree and 5.6% had a university diploma. (2) 38.9% of users worked in the service sector, 27.8% in architecture and engineering, 11.1% each in law and social sciences or health and social services. There were 5.6% of users each working in the arts and humanities or sciences and information technology. At the time of the study, 10 (16.7%) users were not in employment, but they had been working at the time they had their implant. Two of the ten (20%) previously unemployed reported that the main reason for job loss was due to excessive sick leave due to medical appointments. (3) Of 60 respondents, 94.2% were satisfied at work. Post-CI, 41.2% of users felt less discrimination at work due to hearing loss. 67.2% of users considered that their interpersonal relationship and sociability in the workplace had improved post-CI.
Issing et al., 2022 [69]	To evaluate the long-term effects of hearing rehabilitation using CI on the quality of life in older patients (≥ 65 years).	(1) QOL: measured by the World Health Organization Quality-of-Life Scale – OLD (WHOQL-OLD) [124]. (2) Autonomy: the ability to live a self-determined, independent life measured by the WHOQL-OLD [124]. (3) Social participation: the ability to participate in social life and social interactions measured by the WHOQL-OLD [124].	Group I included individuals 1–3 years post-CI, Group II included individuals 4–6 years post-CI, and Group III included individuals 7–10 years post-CI. (1) QoL mean ± SD for group I was 67.9 ± 11.1, for group II it was 69.4 ± 10.5, and for group III it was 65.7 ± 11.4. (2) For autonomy, group I scored 74.1 ± 15.8, group II scored 71.7 ± 16.8, and group III scored 68.1 ± 19.1. (3) For social participation, group I scored 67.1 ± 17.5, group II scored 72.0 ± 11.3, and group III scored 65.9 ± 17.6.
Issing et al., 2020 [67]	To determine the effects of CI hearing rehabilitation on quality of life in older patients (≥ 65 years).	(1) QOL: measured by the WHOQL-OLD [124]. (2) Autonomy: the ability to live a self-determined, independent life measured by the WHOQL-OLD [124]. (3) Social participation: the ability to participate in social life and social interactions measured by the WHOQL-OLD [124].	(1) The total QoL score in the pre-operative period was 60.0 ± 15.7 that changed to 58.5 ± 13.6 at initial fitting of the CI (1 month post-CI) and to 66.8 ± 12.2 six months post-CI. (2) The mean ± SD score of autonomy changed from 63.2 ± 17.6 pre-operatively to 61.4 ± 17 at initial fitting (1 month post-CI) and to 65.3 ± 15 6 months post-CI. (3) The mean ± SD score of social participation changed from 61.4 ± 21.0 pre-operatively to 59.9 ± 18.0 at the initial fitting (1 month post-CI) and 70.6 ± 13.6 6 months post-CI.
Kos et al., 2007 [55]	To verify whether CI helped profoundly deaf adults to maintain or even to develop their professional occupations, and to identify other elements that may contribute to or, on the contrary, impede such patients’ professional success.	Pre- and post-CI. (1) Employment history. (2) Training or developing non-professional skills.	(1) Pre-CI, 34 (57%) of 60 were professionally active, of which 29 (85%) remained active and 5 (15%) became inactive post-CI, due to bilateral vestibular deficit (n = 2), workplace downsizing (n = 2) and retirement (n = 1). Of the 29 who remained active, 4 (14%) achieved positive development in their careers, including 2 promoted to management positions and 2 moved to jobs requiring better skills. Of the 26 (43%) of 60 who were professionally inactive prior to CI, all remained inactive post-CI. Six stated that they had searched intensively for a job but were not successful. (2) Of the 34 patients who were professionally active prior to CI, 10 (29%) developed new non-professional activities such as playing the piano, undertaking a language course, secretarial work for charity institutions post-CI. Of the 26 patients who were professionally inactive prior to CI, none developed new skills.
Krabbe et al., 2000 [99]	To assess the effect of CI use on the perceived health status of adults with profound sensorineural hearing loss.	At least 1-year post-CI. (1) HRQoL: measured by NCIQ [113], SF-36 [108] and HUI-2 [125] (2) Social interaction (self-esteem and activity). (3) Cognition.	The mean ± SD of CI use was 5 ± 2.8 years and the duration of hearing loss for CI users was 13 ± 12 years. (1) The mean ± SD change between pre- and post-CI for HRQoL was significant: Physical functioning (− 3.7 ± 10.5), social functioning (26.5 ± 27.3), role functioning (19.9 ± 40.2) and mental health (15.8 ± 19.9). (2) Social interaction improved from 52.1 ± 17.2 pre-CI to 71.9 ± 14.5 post-CI. Self-esteem domain improved from 42.0 ± 19.6 pre-CI to 66.7 ± 16.4 post-CI. Activity domain improved from 49.0 ± 21.0 pre-CI to 72.9 ± 15.9 post-CI. (3) Cognition changed from 93.2 ± 13.8 pre-CI to 95.5 ± 13.7 post-CI.
Lachowska et al., 2013 [87]	To assess the benefits of CI in elderly patients (≥ 60 years).	Autonomy: assessed by the benefits of CI on everyday life activities at 3 to 12 months pos- CI. After the follow-up appointments, users were interviewed.	The mean follow-up time was 2.34 years. One CI user had stopped using the device after 1.5 years. Most users had auditory rehabilitation prior to the survey. Users reported the CI helped them to hear sounds, to better communication and resulted in improved contact with household members, relatives, and friends that ultimately improved autonomy in everyday life. Users also reported the CI enabled them to communicate with unfamiliar people with a little to no help from lip-reading in noisy environments.
Looi et al., 2011 [74]	To investigate the effect of CI on QoL for adult recipients; and to determine which aspects of life that these changes are most noticed.	(1) QoL: measured by the NCIQ [113]. (2) Experience at work or during education. (3) Social participation: measured by self-esteem, activity limitation and social interaction.	The mean ± SD duration on hearing loss was 32.7 ± 18.4 years for the CI group and 29.8 ± 17.6 years for waiting list (WL) group. The mean duration of CI use was 4.1 ± 4.4 years and 24.10 ± 14.6 years on hearing aid for the WL group. (1) In CI group, the overall mean ± SD of QoL was 69.97 ± 15.54 compared to 41.24 ± 13.88 in the WL group. In the subdomains of QoL, the mean rating for the CI group compared to the WL group was 73.00 ± 18.87 vs. 36.79 ± 16.88 for social interaction, 70.63 ± 21.07 vs. 38.35 ± 19.78 for activity limitations and 64.97 ± 18.22 vs. 42.67 ± 18.74 for self-esteem. (2) CI was associated with the broadening of employment opportunities, and improved job satisfaction and leisure time activities. Difficulties in work and education caused by hearing loss were reported by 51% in the WL group and 5% in the CI group. (3) 74% of CI users felt their self-esteem improved and 69% of CI users felt their social interaction improved post-CI.
Maki-Torkko et al., 2015 [105]	To examine pre-operative expectations and post-operative experiences related to CI in recipients and their significant others.	(1) Experience of participation in work. (2) Views on autonomy: defined as the ability to communicate with others or manage social situation without fear or relying on someone taking the role. (3) Experience of social participation.	The mean ± SD time of device use was 4.9 ± 3.8 years. (1) 50% of CI users were retired from work prior to the study. There was a feeling of improved relations in the workplace and increased perceived self-value post-CI compared to pre-CI. (2) Post-CI, users reported experiencing autonomy in social situations. Users indicated that doing tasks just like anybody else and performing more involved tasks that were too difficult pre-CI. (3) Users reported that CI had improved participation and involvement in social events. There were positive improvements in social exchange such as being able to listen to significant others and having conversations with families or close friends.
Marschark et al., 2018 [100]	To examined relations among social maturity, executive function (EF), CI use, self-efficacy and communication skills among deaf university students.	Executive function (cognitive and academic abilities) measured by the Learning, Executive, and Attention Functioning (LEAF) scale [126, 127].	The mean ± SD of attention domain for CI users was 5.09 ± 3.11 compared to non-users (4.36 ± 2.40). Learning abilities for CI users were mathematics (5.60 ± 3.65), reading (3.89 ± 2.86) and writing (4.72 ± 3.28). Non-CI learning abilities: mathematics (6.18 ± 3.65), reading (3.60 ± 2.56) and writing (3.47 ± 2.72).
Mertens et al., 2021 [61]	To determine the effect of CI on the cognitive evolution in older adults with severe or profound hearing impairment.	Measurements were taken at pre-CI and 14 months post-CI. (1) Cognition: measured by changes in total score on the RBANS-H [112]. (2) Perceived hearing disability: measured by changes in total score of the SSQ12 [116] ranging from 0 to 10, with a lower score indicating a higher degree of perceived hearing disability. (3) Depression and anxiety: measured by the HADS [128].	(1) The mean change in total of RBANS-H score from baseline to 14 months post-CI was 9.1 in the CI group and 4.9 in the non-CI group. (2) The mean change in SSQ12 score from pre-CI to 14 months post-CI was significantly greater in the CI group than in the non-CI group. (3) There was no significant change in depression or anxiety from pre-CI to 14 months post-CI in either the CI group or non-CI group.
Mo et al., 2004 [59]	To compare adult CI patients with two different groups of severely to profoundly deafened adults in a cross-sectional study by using a generic HRQOL measure in addition to a depression and anxiety measure and two disease-specific instruments.	(1) HRQoL: measured by the Patient Quality of Life Form (PQLF) [129, 130], the Index Relative Questionnaire Form (IRQF) [129, 130] and the Short Form 36 (SF-36) [131]. (2) Work, safety and welfare: measured by the Index Relative Questionnaire Form (IRQF) [129, 130]. (3) Anxiety and depression: measured by the Hopkins Symptom Checklist 25 Items (HSCL-25) [132] to assess the presence and intensity symptoms over the previous week.	The mean ± SD duration between CI and evaluation was 6.3 ± 4.0 years (range 0.8 to 14.5 years). While the time interval between evaluation of hearing to investigation in the non-CI group was 5.1 ± 3.7 years. The non-CI group was divided into Group A (i.e. met criteria for CI, but did not receive a CI) and Group B (i.e. rejected for CI, as hearing levels were good). (1) The mean total PQLF score was significantly higher for the CI group (3.53) compared with non-CI group B (3.28), but not the non-CI group A (3.37) or hearing aid (HA) group (3.57). The mean total IRQF score was significantly higher for the CI group (3.78) compared to non-CI subgroup A (3.53) and non-CI group B (3.52), but not the HA group (3.73). There was significant difference in mean score for mental health between the CI group and non-CI subgroup A (82.8 vs. 71.5). The CI group had significantly better mean score for role emotional than the HA group (84.4 vs. 72.2). (2) There was no significant difference in relation with close friends, work, hobbies and activities, or safety and welfare between CI group and non-CI groups or HA group. (3) Mean total HSCL-25 score was significantly lower for CI group (1.39) compared with non-CI group A (1.67), but not the non-CI group B (1.39) or the HA group (1.40).
Mo et al., 2005 [60]	To evaluate changes in QoL, anxiety, and depression post-CI in adults.	All outcomes were reported pre-CI and 12 to 15 months post-CI. (1) HRQoL: measured by the PQLF [129, 130], IRQF [129, 130] and SF-36 [131]. (2) Work, and safety and welfare: measured by mean score change in PQLF and IRQF sub-domain scores, respectively, pre and post CI surgery. (3) Anxiety and depression: measured by the presence and intensity of symptoms in the previous week and data were collected by using HSCL-25 [133].	The mean duration of hearing loss pre-CI was 8.5 ± 10.3 years and all users had post lingually hearing loss. (1) The overall mean ± SD PQLF score was 2.94 ± 0.54 for pre-CI and 3.56 ± 0.44 for post-CI. The mean ± SD difference between pre-and post-CI was 0.62 ± 0.47. There was significant increase in mean ± SD scores on the SF-36 general health category from pre-CI (72.6 ± 21.6) to post-CI (79.8 ± 21.4). (2) Compared to pre-CI, there was no significant change in mean scores for categories *hobbies* or *work* or *safety* or *welfare* post-CI. (3) Compared to pre-CI, there was significant decrease in the degree of anxiety and depression at 12 months post-CI.
Monteiro et al., 2012 [58]	To determine the economic impact of profound deafness and subsequent effects of unilateral CI.	(1) Employment: change in status of employment post-CI. (2) Income: the Government of Canada’s Human Resources and Skills Development website [134] was used to estimate the personal annual income prior to and post implantation.	(1) The rate of employment decreased by 5% between first time hearing loss diagnosis and initial assessment for CI eligibility (45.3% vs. 40.3%). Almost half (51.1%) of the CI users were employed post-CI, which was a 10.8% increase from initial assessment for CI. There were 126 (34.2%) CI users who changed their employment status post-CI, of which 77.8% reported positive changes while 22.2% reported negative changes. Of those who reported positive changes, 83.8% of CI users perceived that the changes were due to CI. (2) The median annual income of CI users increased from CAD $30,432 at initial assessment for CI to CAD $42,672 post-CI.
O’Neill et al., 2021 [101]	To assess the listening behaviour and social engagement of CI users and normal-hearing adults in daily life and relate these actions to objective hearing outcomes.	At least 2 years post-CI. (1) Social engagement measured by ecological momentary assessment (EMAs) and completed via smartphone app. (2) Work.	The mean duration of hearing loss prior to implantation was 11 ± 10.5 years and used an implant for 11.9 ± 6.2 years. (1) On average, poorer CI users (speech understanding < 40%) spent 60% of their time alone and only interacted or were around others about 40% of their time. Good CI users (speech understanding > 40%) reported being alone 50% of the time and with others 50% of the time. CI users spend most of their time in listening and social situations they find to be “not difficult.” (2) The proportion of users not working or retired was 44% for good CI users and 56% for poor CI users.
Park et al., 2011 [95]	To determine whether preoperative use of hearing aids correlates with improvement in speech recognition and perceived quality of life post-CI.	Approximately 1-year post-CI. Perceived hearing disability measured by Hearing Handicap Inventory (HHI) [135] post-CI.	CI reduced overall HHI score by approximately 45% (p < 0.01). Pre-CI, the mean and standard error of HHI score on the aided side ear was (78.1 ± 3.3) and on nonaided side was (77.1 ± 3.1). Post-CI, the HHI scores were (41.8 ± 2.5) on the CI aided side and (48.7 ± 4.3) on the nonaided side.
Rembar et al., 2009 [56]	To gain a deeper insight into the effects of CI on recipients’ lives, as perceived by the recipients themselves.	(1) Views on employment, social participation, and autonomy. (2) Experience of education. (3) Perceived hearing disability was assessed as experience of being more confident, level of energy and bodily aspect.	Users had hearing loss for the mean ± SD duration of 31.8 ± 13.4 years. The mean ± SD time interval between CI surgery and study participation was 2.1 ± 1.5 years. (1) Most users were either working (51.4%) or retired (29.7%), while 16.2% were receiving social welfare. Users reported improvements in social participation, communication, and interpersonal relationships post-CI. (2) Compared to pre-CI, users indicated improvements in education and employment status post-CI (e.g. better at the job). (3) Users perceived that CI improved their self-confidence (e.g., having CI was perceived as like having a new life).
Ross et al., 2007 [45]	To map the experiences of adults and their hearing partners living with deafness; and the changes brought about by CI recipients.	(1) Views on social participation. (2) Experience of employment.	The average duration of CI use was 2.6 years. (1) Users reported improvement in social participation (i.e. social interaction). Pre-CI, users experienced a lack of self-confidence that gradually improved post-CI. (2) Some users reported experiencing challenges in their workplace that related to employers’ expectations post-CI (e.g. a lack of understanding of gradual adjustment to improve hearing).
Saxon et al., 2001 [102]	To determine the impact of CI on the job functioning of adults with profound hearing loss.	Job functioning: measured by the Abbreviated Profile of Hearing Aid Benefit Questionnaire (APHAB) [136] pre- and at least 6 months post-CI.	The duration of hearing loss ranged from 3 to over 10 years and implant use ranged from six months to over 3 years. Overall, self-reported ratings of CI users and their work supervisors of work and social experiences improved post-CI compared to pre-CI.
Sonnet et al., 2017 [53]	To evaluate QoL and cognitive function in elderly patients with cochlear implants relative to auditory improvement, using geriatric validated scales.	Assessments were conducted pre- and at 6- and 12-months post-CI. (1) Autonomy: measured by the Instrumental Activity of Daily Living (I-ADL) [137, 138] on four domains such as telephone use, transportation, medication, and domestic finances. (2) QoL: assessed by using WHOQoL-OLD [139]. (3) Cognition: assessed by Mini-Mental State Evaluation (MMSE) [140] on domains such as executive functions, memory and language abilities. (4) Depression: recorded by using the HDS [141].	On average, users had hearing loss for 17 years and worn hearing aids at least for 15 years. (1) Mean ± SD autonomy score significantly increased from pre-CI (result not shown) to 12 months post-CI (0.94 ± 0.10), but not at 6 months post-CI (0.84 ± 0.22) (2) The mean ± SD score of sensory abilities increased from 7.0 ± 2.0 pre-CI to 12.9 ± 4.7 at 6 months post-CI and to 13.3 ± 3.3 at 12 months post-CI. (3) Mean ± SD scores changed from 27.1 ± 2.1 pre-CI to 26.0 ± 3.0 at 6-month to 27.7 ± 1.6 at 12 months post-CI. When measured in percentage, the mean ± SD was 94 ± 5 pre-CI, 88 ± 9.0 at 6-month and 94 ± 4.0 at 12-month post-CI. (4) Depression did not change significantly from pre-CI during the follow-up periods.
Spencer et al., 2012 [47]	To replicate previous findings and to provide additional educational, vocational, and living status information for the first two cohorts of CI recipients.	(1) Education or vocational measured by the Living Status Questionnaire. (2) Employment. (3) QoL measured by SWLS [118] as life satisfaction.	(1) The proportion of CI users with high school graduate was higher compared with that of the general population in the U.S. Census (34% vs. 27%, respectively). There was higher proportion of college level educational attainment for CI users compared with general population (32% vs. 24%, respectively). (2) Types of employment included business administration, agriculture, health care, and service. (3) CI users reported a high level of satisfaction with life (mean ± SD SWLS score = 27.54 ± 4.52).
Vieira et al., 2018 [46]	To understand the benefits of CI in adulthood under the perspective of users.	Semi-structured interviews pre- and post-CI. (1) Highest level of education. (2) Views on work. (3) Views on autonomy. (4) Experience of social participation.	The mean duration since CI was 3.9 years. (1) Users’ educational status varied: 1 user had not completed middle school, 8 users completed middle or high school, 4 had college or university degrees and 3 had incomplete university degrees. (2) Users indicated that having a CI helped them to work, resulted in better interaction in conversations, and better performance in the workplace. (3) CI helped users to be less dependent on other people for communication. (4) Post-CI, more efficient communication and social interaction improved. Feelings of self-isolation reduced post-CI compared to pre-CI.
Volter et al., 2018 [54]	To identify the impact of hearing rehabilitation via CI on cognitive decline among the aging population.	Outcomes were assessed pre- CI and at 6- and 12-month post-CI. (1) Cognition: measured by a neurocognitive test battery. (2) QoL: measured by the NCIQ [113]. (3) Autonomy and (4) Social participation were collected using WHOQoL-OLD [142].	The average time between the first hearing aid use and CI was 25.4 years. (1) Cognition improved from pre-CI to 6-month post-CI but remained stable from 6 to 12 months post-CI. (2) The mean ± SD of general QoL changed from 72.52 ± 7.37 pre-CI to 75.41 ± 7.56 at 6 months post-CI. At 6 months post-CI, HRQoL significantly improved across all domains from pre-CI. At 12 months post-CI, the HRQoL scores remained stable across all domains. (3) Autonomy increased significantly from 15.1 ± 2.83 pre-CI to 15.97 ± 1.72 at 6 months post-CI and remained stable at 12 months post-CI. (4) Compared to pre-CI, at 6 months post-CI, activity limitation decreased by 17.6%, social interaction increased by 17.8%, and self-esteem increased by 13.3%, with no significant changes in level of activities, social participation, or intimacy.

Of the 21 studies that reported on work or employment, the majority (85.7%, n = 18) of studies found positive improvements on work or employment status post-CI. However, three studies (14.3%) did not find an additional benefit of CI on work or employment status. For example, one study conducted by Ross and Lyon [45], found CI users experienced difficulties in their workplace as employer expectations of hearing improvement post-CI were unrealistic.

Of the 15 studies that reported on social participation, improvement in social participation was observed in 14 (93.3%) studies post-CI. Only one study found no additional benefits in social participation post-CI [51]. The Chapman et al. 2017 [51] found that the CI cohort showed increased feelings of being limited due to their hearing loss, and that they participated less in mainstream organisational activities than the non-CI group. However, after categorising age into ≤ 25 and > 25 years, the authors found no statistical difference by age in participation in mainstream organisational activities. Also, the CI users in the older age group were found to socialise more with their hearing friends compared with non-users.

Independence or autonomy was reported in nine studies. Eight (88.9%) studies found substantial improvement in independence or autonomy following a CI. Only one study found no improvement in independence or autonomy measured by a subscale of quality of life [52]. Of the studies that found improvements in autonomy post-CI, Sonnet et al. 2017 [53], found improvement at 12-months post-CI. While another study [54] found a significant difference between pre- and post-implantation on autonomy at 6 months post-implantation, but this result did not persist at the 12-month follow-up.

Education or training was measured in nine studies. All studies that measured education or training found benefits post-CI. For example, Goh et al. 2016, found that 76% of CI users reported that the CI enabled them to access learning opportunities and gain tertiary qualifications [44]. Another study found that CI helped adults in retaining and developing professional abilities, for example two CI users were promoted to management positions and two moved to jobs requiring a higher level of skills [55].

Six studies measured perceived hearing disability. Five of the six studies found some improvements among respondents post-CI. For instance, one qualitative study [56] indicated participants perceived that the CI provided them a ‘new life’. CI was found to be associated with type of identity, such as deaf or hearing identity, type and quality of friendships, social activities, and feelings of limitation previously attributed to hearing loss [51].

Three studies reported on income and two on safety and welfare benefits. The three studies that reported on income, all found increases in the recipient’s income level post-CI. For example, one study [57] found a 31% increase in income bracket after a mean 6.6 years post-CI. Montero and colleagues [58] also found a significant increase in median annual income post-CI compared with preimplantation (CAD $42,672 vs. CAD $30,432). However, none of the identified studies found a positive change in welfare benefits. For instance, Mo et al. 2004 found no significant difference between CI and non-CI users in terms of safety, and welfare [59]. Again, the authors found that after 12 and 15 months post-CI, welfare and safety were not significantly improved [60].

### Communication, anxiety, depression, quality of life and cognition

Of the 35 studies that reported on societal outcomes, ability to communicate was reported in 27 (77.1%), QoL in 15 (42.9%), and six (17.1%) studies each reported anxiety or depression and cognition. Of the studies that reported on communication abilities and QoL, all found improvement post-CI. Four of the six studies that reported on anxiety or depression found a reduced level of anxiety or depression post-CI. For example, Mo et al. 2005 [60] found the reduced mean score of anxiety and depression between pre- and post-implantation (-0.10 vs. -0.19). While two of the six studies found no change for anxiety or depression post-CI [53, 61]. There were mixed results on the effects of CI on anxiety, depression and cognitive function over time. For instance, Claes et al. 2018 found a decreased level of anxiety and depression at six months post-CI, however, the decrease was not sustained at a 12 month follow-up [62]. In contrast, another study found a decreased level of anxiety and depression after 12 months and 15 months post-CI [60] that was associated with gain in QoL. On the other hand, in the remaining study at 6 and 12 months post-CI, anxiety, depression levels and cognitive function remained stable [53].

### Quality assessment

The overall quality of included studies was deemed low. None of the included studies had a low risk of bias due to inadequate methods to minimise the effects of confounding factors at design and analysis stage as defined by the CASP criteria. Few studies (36.4%, n = 16) scored ‘Yes’ for questions related to minimising the effects of confounding factors (see Additional file 2).

## Discussion

This systematic review has synthesised evidence found in 44 studies regarding health service utilisation and social outcomes in adult CI users. The review identified limited research on health service utilisation post-CI. A systematic review that incorporated quantitative pooling of prospective studies to produce overall effect size is imperative. Despite a small number of studies examined health service utilisation, more than half found benefits of a CI. Most included studies (77.1%) have reported improvements for at least one social outcome post-CI.

Relatively small number of CI users who experienced complications required CI revision surgery. The review found that device failure (soft and hard) and medical-related predominantly skin flap-related infections were common reasons for the revision surgery. The current finding supports the need to maintain long-term follow-up post-CI to identify and manage any potential complications. Also, this study supports the importance of counselling for users about realistic expectations post-CI surgery. Prior research recommends that CI users receive a lifetime follow-up to identify and monitor any long-term complications [63, 64].

The current review found that CI users took prescribed medications for a lower number of illnesses than individuals on a wait list for a CI [50]. Similarly, prior studies have indicated that wearing hearing aids or cochlear implants improved communication abilities that further translated into improved health conditions and reduced unnecessary self-medication [19, 65]. However, it has been shown that waiting for medical intervention increases anxiety, depression and may reduce QoL [66]. As such, an individual’s position on a waiting list for CI may lead to negative emotions and associated physiological responses [50].

The current review found substantial improvement in work or employment status post-CI. These findings are consistent with a prior review that indicated evidence of improvement in work performance and employment status post-CI [36]. Only a few studies did not find an additional benefit of CI on work or employment. For instance, CI users reported challenges in their workplace because employers expected that CI can fully restore their normal hearing [45]. Further research that examines employers’ knowledge and expectations post-CI in the workplace may be of benefit.

Improvements were observed in social participation and in autonomy in almost all studies post-CI. Cochlea implant was associated with improved quality of life and speech perception which led to a demonstrated improvement in social participation [54, 67, 68]. CI was also found to improve independence in the adult population. Improvements in QoL may primarily be responsible for the increased feelings of autonomy or independence [69]. Similarly, a scoping review that examined the effects of CI on autonomy, participation and work found similar improvements post-CI [36]. The current review identified inconsistent definitions for both social participation and autonomy as well as inconsistency in the tools used to assess these constructs across studies. Within the included studies, there was an overlap between the examination of social participation and interrelated definitions, such as self-esteem, independence, activity limitations or QoL. Likewise, there were differences in the measurement of autonomy or independence across studies. Although the current review was inclusive of all definitions, further research is suggested to develop validated definitions for social participation and for autonomy or independence for CI users.

All studies that measured effects of CI on education or training found benefits post-CI. Compared with the general population, young adult CI users had a higher rate of attendance at post-secondary education programs and also reported being able to achieve their academic and personal goals [70]. Similarly, a review of five randomised controlled trials in adults who had received a hearing aid found that hearing aids were viewed as having improved educational opportunities [71].

Three studies reported on a CI recipient’s income [57, 58, 72] and all found a higher income level post-CI. However, none of the studies reporting on welfare found additional benefits post-CI. For example, Mo et al. 2004 compared CI with non-CI users and found no significant differences in terms of welfare or safety [59], even at 12 and 15 months post-CI [60]. Large scale and well-designed epidemiological studies are needed to examine the long-term association of CI on income or welfare benefits.

Studies that examined QoL found substantial improvement post-CI using different assessment tools [52–54, 59, 60, 62, 67–69, 73–76]. The current review findings are in line with previous reviews that found improvement in QoL post-CI [77–79]. For example, Andries et al. 2021, found improvements in health-related quality of life (HRQoL) pre- and post-CI. Andries et al. study examined HRQoL in older adults only, but the current review examined general QoL and HRQoL, health service utilisation and other social outcomes both in younger and older adults. It seems that CI users felt they confidently communicated which resulted in improvements in health-related or general QoL. Prior research has also indicated improved communicative ability through rehabilitation resulted in improved QoL in older adults [80, 81].

The current review found a link between CI and anxiety or depression, with most included studies identifying improvements [59, 60, 62]. The current review is consistent with prior research which found improvements in internalising mental health conditions [82–86]. Improvements in mental health from pre-CI were seen in the first 6 months but diminished after a long-term follow-up at 12 months post-CI in a study of 20 older adults [62]. Several factors could explain these results including users may relate their expectations to unrealistic outcomes, such as device limitations and its maximum benefits post-CI [87].

In this review, CI was found to improve cognitive functions such as immediate memory, attention, and delayed memory subdomains at 12 months post-CI. In older adults, CI was associated with improved cognitive functions mainly by improving the attention domain [54, 61, 62]. These results corroborate previous research findings that found CI was associated with improvement in cognitive function in older adults [80, 82, 88, 89].

### Strengths and limitations

The strengths of this review were that it followed the PRISMA guidelines, the search strategy was developed by consulting a university librarian, and dual screening and data extraction was conducted. Despite these strengths, there are limitations for this review. First, most studies reported results based on small sample sizes, which made it difficult to generalise findings to larger populations. Second, the validity of questionnaires for measuring outcomes such as social participation, autonomy or perceived hearing disability in included studies were not known. Studies have used different measurement tools for the same social outcomes, this may lead to variation in patient outcomes or inaccuracy of measured outcomes. Third, post-operative complications of care may not have been observed, or may be underreported, in the included studies. For example, these studies may have only been conducted with CI users who use their cochlear implant, limiting the knowledge that could be gained from CI users who may have stopped using their devices.

## Conclusions

Despite identifying small body of evidence regarding health service utilisation, this review found benefits of CI in improving adults’ health service utilisation and social outcomes. Improvement in hearing and communication ability was shown to enhance social interactions and working life, and also to support independence in everyday life. However, the review highlights the need for large scale and well-designed epidemiological studies to measure health and social outcomes.

### Electronic supplementary material

Below is the link to the electronic supplementary material.

Supplementary Material 1

Supplementary Material 2

## Data Availability

All data generated during this study are included in this systematic review and its supplementary information files.

## Abbreviations

CI: Cochlear implant
HRQoL: Health related quality of life
NR: Not reported
QoL: Quality of life
SD: Standard deviation
WHO: World Health Organization
