# Supplementary material for: Cochlear implantation impact on health service utilisation and social outcomes: a systematic review
Source: BMC Health Serv Res. 2023 Aug 30;23:929. doi: 10.1186/s12913-023-09900-y (PMC10468908; doi:10.1186/s12913-023-09900-y)
Supplement: Supplementary file 1 — Supplementary Material 1 [file 12913_2023_9900_MOESM1_ESM.docx]

### **Full search strategy**

### **Medline and PsychINFO**

Cochlear Implantation/

Cochlear Implant/

health services/ or community health service/ or exp emergency medical services/ or health services for persons with disabilities/ or health services for the aged/ or health services, indigenous/ or health services misuse/ or medical overuse/ or overtreatment/ or unnecessary procedures/ or mental health services/ or community mental health services/ or exp counselling/ or personal health services/ or exp pharmaceutical services/ or exp rehabilitation/ or healthcare utili?ation.tw. or health service use*.tw. or general practitioner visit*.tw. or primary care*.tw. or dental service*.tw. or hospital.tw. or hospital admission*.tw. or palliative service*.tw. or hospitalization*.tw. or specialist visit*.tw. or hospital readmission*.tw. or emergency department presentation*.tw. or oral health service*.tw. or allied health service*.tw. or consultation/ or consultation service*.tw. or care complication*.tw. or (societal or social) or (education or training) or (academic performance).tw. or disability/ or social welfare*.tw. or social housing*.tw. or welfare benefit*.tw. or occupation/ or work/ or employment/ or (income or income level).tw. or personal relationship*.tw. or self-esteem*.tw. or (social isolation or loneliness).tw. or depression*.tw. or cognitive*.tw. or communication*.tw. or quality of life/ or feeling inadequacy*.tw. or (independence or autonomy).tw. or social participation*.tw. or (societal or social outcome).tw. or (apply for higher education).tw.

### **Scopus**

( "cochlear implant*") AND ("health service use*" OR " medical service*" OR "emergency medical service*" OR "health service for the aged*" OR "hospital admission*" OR "hospitalization*" OR "specialist visit*" OR "general practitioner visit*" OR "health service*" OR "palliative service*" OR "emergency department presentation*" OR "allied health service*" OR "consultation*" OR "care complication*" OR "oral health service*" OR "pharmaceutical use*" OR "primary care*" OR "dental service*") OR ("cochlear implant*") AND ("societal outcomes*" OR "education*" OR "training*" OR "academic performance*" OR "disability*" OR "social welfare*" OR "social housing*" OR "welfare benefits*" OR "occupation" OR "work" OR " employment" OR "income" OR "personal relationship*" OR "independence*" OR "autonomy*" OR "social participation*" OR "self-esteem*" OR "social isolation*" OR "loneliness*" OR "depression*" OR "cognitive*" OR "communication*" OR "quality of life" OR "feeling inadequacy" OR "apply for higher education*")

**ERIC**

( "cochlear implant*") AND ("health service use*" OR " medical service*" OR "emergency medical service*" OR "health service for the aged*" OR "hospital admission*" OR "hospitalization*" OR "specialist visit*" OR "general practitioner visit*" OR "health service*" OR "palliative service*" OR "emergency department presentation*" OR "allied health service*" OR "consultation*" OR "care complication*" OR "oral health service*" OR "pharmaceutical use*" OR "primary care*" OR "dental service*") OR ("cochlear implant*") AND ("societal outcomes*" OR "education*" OR "training*" OR "academic performance*" OR "disability*" OR "social welfare*" OR "social housing*" OR "welfare benefits*" OR "occupation" OR "work" OR " employment" OR "income" OR "personal relationship*" OR "independence*" OR "autonomy*" OR "social participation*" OR "self-esteem*" OR "social isolation*" OR "loneliness*" OR "depression*" OR "cognitive*" OR "communication*" OR "quality of life" OR "feeling inadequacy" OR "apply for higher education*")
