# Supplementary material for: Cochlear implantation impact on health service utilisation and social outcomes: a systematic review
Source: BMC Health Serv Res. 2023 Aug 30;23:929. doi: 10.1186/s12913-023-09900-y (PMC10468908; doi:10.1186/s12913-023-09900-y)
Supplement: Supplementary file 2 — Supplementary Material 2 [file 12913_2023_9900_MOESM2_ESM.docx]

Quality assessment results for included studies

**Table s1** Quality assessment using the CASP checklist for cohort studies

| **Authors** | **Q1** | **Q2** | **Q3** | **Q4** | **Q5a** | **Q5b** | **Q6a** | **Q6b** | **Q8** | **Q9** | **Q10** | **Q11** | **Q12** |
| --- | --- | --- | --- | --- | --- | --- | --- | --- | --- | --- | --- | --- | --- |
| Aldhafeeri et al, 2021 [[90](#_ENREF_90)] | Y | Y | Y | N | N | N | Y | Y | C | Y | Y | Y | Y |
| Chen et al, 2022 [[91](#_ENREF_91)] | Y | Y | Y | N | N | N | Y | Y | Y | Y | Y | Y | Y |
| Claes et al, 2018 [[62](#_ENREF_62)] | Y | Y | Y | Y | N | C | Y | Y | Y | Y | Y | Y | Y |
| Czerniejewska-Wolska et al, 2015 [[68](#_ENREF_68)] | Y | Y | Y | Y | N | N | Y | Y | C | Y | Y | Y | Y |
| Gumus et al, 2021 [[92](#_ENREF_92)] | Y | Y | Y | Y | N | Y | Y | Y | C | Y | Y | Y | Y |
| Harkonen et al, 2015 [[75](#_ENREF_75)] | Y | Y | Y | Y | N | N | Y | Y | Y | Y | Y | Y | Y |
| Hawthorne et al, 2004 [[76](#_ENREF_76)] | Y | Y | Y | Y | N | N | C | Y | Y | Y | Y | Y | Y |
| Huarte et al, 2017 [[93](#_ENREF_93)] | Y | Y | Y | Y | N | N | C | Y | C | Y | Y | Y | Y |
| Issing et al, 2022 [[69](#_ENREF_69)] | Y | Y | Y | Y | N | Y | Y | Y | Y | Y | Y | Y | Y |
| Issing et al, 2020 [[67](#_ENREF_67)] | Y | Y | Y | Y | N | Y | Y | Y | Y | Y | Y | Y | Y |
| Kay-Rivest et al, 2022 [[94](#_ENREF_94)] | Y | Y | Y | Y | Y | N | N | Y | C | Y | Y | Y | Y |
| Lachowska et al, 2013 [[87](#_ENREF_87)] | Y | Y | Y | N | N | N | Y | Y | C | Y | Y | Y | Y |
| Mertens et al, 2021 [[61](#_ENREF_61)] | Y | Y | Y | Y | Y | Y | Y | Y | Y | Y | Y | Y | Y |
| Mo et al, 2005 [[60](#_ENREF_60)] | Y | Y | Y | Y | N | N | Y | Y | Y | Y | Y | Y | Y |
| Monteiro et al, 2012 [[58](#_ENREF_58)] | Y | Y | Y | Y | N | N | N | Y | C | Y | Y | Y | Y |
| Park et al, 2011 [[95](#_ENREF_95)] | Y | Y | Y | Y | N | N | N | Y | Y | Y | Y | Y | Y |
| Raymond et al, 2020 [[48](#_ENREF_48)] | Y | Y | Y | Y | N | N | N | Y | Y | Y | Y | Y | Y |
| Sonnet et al, 2017 [[53](#_ENREF_53)] | Y | Y | Y | Y | Y | Y | Y | Y | Y | Y | Y | Y | Y |
| Sorrentino et al, 2016 [[96](#_ENREF_96)] | Y | Y | Y | Y | N | N | C | Y | C | Y | Y | Y | Y |
| Volter et al, 2018 [[54](#_ENREF_54)] | Y | Y | Y | N | N | N | Y | Y | Y | Y | Y | Y | Y |

Y- Yes; N- No; C- Can’t tell; Q- question; Answer for “What are the results of this study?” has been presented in Tables 2 and 3 in the manuscript, thus not included in this Table s1.

*CASP Cohort studies appraisal checklist questions*

| 1. Did the study address a clearly focussed issue? | 6b. Was the follow up of subjects long enough? |
| --- | --- |
| 2. Was the cohort recruited in an acceptable way? | 8. How precise are the results? |
| 3. Was the exposure accurately measured to minimise bias? | 9. Do you believe the results? |
| 4. Was the outcome accurately measured to minimise bias? | 10. Can the results be applied to the local population? |
| 5a. Have the authors identified all important confounding factors? | 11. Do the results of this study fit with other available evidence? |
| 5b. Have they considered the confounding factors in the design and/or analysis? | 12. Does the study have implications for practice? |
| 6a. Was the follow up of subjects complete enough? |  |

**Table s2** Quality assessment using the CASP checklist for cross sectional studies

| **Authors** | **Q1** | **Q2** | **Q3** | **Q4** | **Q5a** | **Q5b** | **Q6** | **Q7** | **Q8** | **Q9** | **Q10** |
| --- | --- | --- | --- | --- | --- | --- | --- | --- | --- | --- | --- |
| Carpenter et al, 2010 [[49](#_ENREF_49)] | Y | Y | Y | Y | N | N | C | Y | Y | Y | Y |
| Chapman et al, 2017 [[51](#_ENREF_51)] | Y | Y | Y | Y | N | N | Y | Y | Y | Y | Y |
| Clinkard et al, 2015 [[57](#_ENREF_57)] | Y | Y | Y | Y | N | N | N | Y | Y | Y | Y |
| Cole et al, 2022 [[97](#_ENREF_97)] | Y | Y | Y | Y | N | Y | Y | Y | Y | Y | Y |
| Fazel et al, 2007 [[98](#_ENREF_98)] | Y | Y | Y | Y | N | N | Y | Y | Y | Y | Y |
| Goh et al, 2016 [[44](#_ENREF_44)] | Y | Y | Y | Y | Y | N | Y | Y | Y | Y | Y |
| Guitar et al, 2013 [[50](#_ENREF_50)] | Y | Y | Y | Y | Y | N | Y | Y | Y | Y | Y |
| Harkonen et al, 2017 [[73](#_ENREF_73)] | Y | Y | Y | Y | N | N | Y | Y | Y | Y | Y |
| Hixon et al, 2017 [[72](#_ENREF_72)] | Y | Y | Y | N | N | N | Y | Y | Y | Y | Y |
| Hogan et al, 2001 [[52](#_ENREF_52)] | Y | Y | Y | Y | Y | N | Y | Y | Y | Y | Y |
| Kos et al, 2007 [[55](#_ENREF_55)] | Y | Y | Y | Y | Y | N | C | Y | Y | Y | Y |
| Krabbe et al, 2000 [[99](#_ENREF_99)] | Y | Y | Y | Y | Y | N | Y | Y | Y | Y | Y |
| Looi et al, 2011 [[74](#_ENREF_74)] | Y | Y | Y | Y | Y | N | Y | Y | Y | Y | Y |
| Marschark et al, 2018 [[100](#_ENREF_100)] | Y | Y | Y | N | N | N | Y | Y | Y | Y | Y |
| Mo et al, 2004 [[59](#_ENREF_59)] | Y | Y | Y | Y | Y | N | Y | Y | Y | Y | Y |
| O’Neill et al, 2021 [[101](#_ENREF_101)] | Y | Y | Y | Y | N | N | Y | Y | Y | Y | Y |
| Spencer et al, 2012 [[47](#_ENREF_47)] | Y | Y | Y | Y | N | N | Y | Y | Y | Y | Y |
| Saxon et al, 2001 [[102](#_ENREF_102)] | Y | Y | Y | N | N | N | N | Y | Y | Y | Y |

Y- Yes; N- No; C- Can’t tell; Q- question; Answer for “What are the result of this study?” has been provided in Tables 2 and 3 in the manuscript, thus not included in this Table s2.

CASP Cross-sectional studies appraisal checklist questions

| 1. Did the study address a clearly focussed issue? |  |
| --- | --- |
| 2. Was the cohort recruited in an acceptable way? | 7. Do you believe the results? |
| 3. Was the exposure accurately measured to minimise bias? | 8. Can the results be applied to the local population? |
| 4. Was the outcome accurately measured to minimise bias? | 9. Do the results of this study fit with other available evidence? |
| 5a. Have the authors identified all important confounding factors? | 10. Does the study have implications for practice? |
| 5b. Have they considered the confounding factors in the design and/or analysis? |  |
| 6. How precise are the results? |  |

**Table s3** Quality assessment using the CASP checklist for qualitative studies

| **Authors** | **Q1** | **Q2** | **Q3** | **Q4** | **Q5** | **Q6** | **Q7** | **Q8** | **Q9** | **Q10** |
| --- | --- | --- | --- | --- | --- | --- | --- | --- | --- | --- |
| Fitzpatrick et al, 2022 [[103](#_ENREF_103)] | Y | Y | Y | Y | Y | N | Y | Y | Y | Y |
| Hogan et al, 2002 [[104](#_ENREF_104)] | Y | Y | Y | N | Y | N | N | Y | Y | Y |
| Maki-Torkko et al, 2015 [[105](#_ENREF_105)] | Y | Y | Y | Y | N | C | Y | Y | Y | Y |
| Rember et al, 2009 [[56](#_ENREF_56)] | Y | Y | N | Y | N | C | Y | N | Y | Y |
| Ross et al, 2007 [[45](#_ENREF_45)] | Y | Y | Y | Y | Y | N | Y | N | Y | Y |
| Vieira et al, 2018 [[46](#_ENREF_46)] | Y | Y | Y | Y | Y | N | Y | Y | Y | Y |

Y- Yes; N- No; C- Can’t tell; Q- question.

CASP Qualitative studies appraisal checklist questions

| 1. Was there a clear statement of the aims of the research? | 8. Was the data analysis sufficiently rigorous? |
| --- | --- |
| 2. Is a qualitative methodology appropriate? | 9. Is there a clear statement of findings? |
| 3. Was the research design appropriate to address the aims of the research? | 10. How valuable is the research? |
| 4. Was the recruitment strategy appropriate to the aims of the research? |  |
| 5. Was the data collected in a way that addressed the research issue? |  |
| 6. Has the relationship between researcher and participants been adequately considered? |  |
| 7. Have ethical issues been taken into consideration? |  |
